# Genome Sequence of *Microbacterium foliorum* Phage KingKamren

**DOI:** 10.17912/micropub.biology.001483

**Published:** 2025-04-10

**Authors:** Alyssa Gleichsner, Kamren Harden, Kathryn Salphine, Jill Sandel, Mikaela Bova, Bella Denapole, Anastasia Godlewski, Samara Acevedo, Alexander Galarneau, Mazon Zales, Gustavia Twumasi, Sophia Voss, Faith Haynes, Rebekah Abdul-Wahhab, Banfy Bu, Abigail Favro, Amen Zergaw, Meherun Maisha, Bian Oliva, Sukhpreet Kaur, Amma Kwatia, Ashley Rufino, Cesia Arzu, Luke Tyrrell, Pamela Pena, Megan Valentine

**Affiliations:** 1 SUNY Plattsburgh, Plattsburgh, New York, United States

## Abstract

We report the discovery and genome sequence of a cluster EK bacteriophage, KingKamren, isolated from a soil sample collected in Plattsburgh, New York using the bacteria *Microbacterium foliorum* , B-24224. Its 54,721 bp genome contains 51 putative genes, 17 of which have predicted functions.

**Figure 1. KingKamren plaques, Gene Content Similarity Comparison, and genome:**
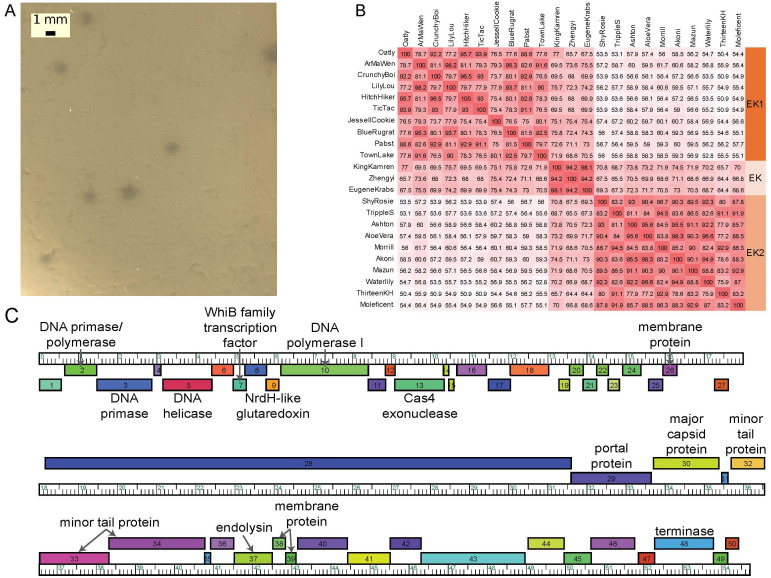
(A) Plaques of phage KingKamren in top agar with *M. foliorum* . (B) Gene Content Similarity map, containing GSC values from the GCS tool (Hirokawa et al., 1998) using random selection of 10 phages from the EK1 and EK2 subclusters and all current EK cluster phages. (C) Genome map for KingKamren, with putative genes presented as colored boxes along a genome ruler, in kilobases. Boxes above and below the ruler represent genes that are transcribed rightwards and leftwards, respectively.

## Description

Bacteriophages are some of the most genetically diverse entities known (Pope et al., 2015). Understanding this diversity has implications for both ecological and health applications (Milhaven et al., 2023; Strathdee et al., 2023). Here we present the genome of a new bacteriophage, KingKamren, which was collected in 2023 from Plattsburgh NY (44.69179 N, 73.46351 W) using the bacterial host *Microbacterium foliorum* , B-24224.

Following standard procedures (Zorawik et al., 2024), approximately 15 cm ^3^ of soil was suspended in 35 mL PYCa medium and shaken at 37°C at 250 rpm for 2 hours followed by centrifugation at 2,000 g and vacuum filtration (0.22 μm filter) of the supernatant. This filtrate was inoculated with *M. foliorum* and incubated at 30°C for 5 days at 250 rpm. An aliquot was spun at 14,000 g, filtered, and plated in PYCa top agar containing *M. foliorum* . After 48 hours, KingKamren formed small, turbid plaques with an average size of 1.17 mm (∓ 0.10 SE) in diameter, as determined through measurements using ImageJ (Schnieder et al., 2012) ( Fig. 1A ) and was purified through two rounds of plating.

DNA was isolated from a lysate (Wizard DNA Clean-up Kit, Promega), prepped for sequencing (NEBNext Ultrall FS Kit), sequenced (Illumina sequencing, v3 reagents) and assembled as described by Russell (Russell, 2018). Sequencing resulted in 2,929,352 single-end 150-bp reads with 4,991-fold coverage. The genome was assembled using Newbler v2.9 (Margulies et al,. 2005) and checked for completeness and genome termini using Consed v29.0 (Gordon et al., 1998). Default settings were used unless otherwise noted. This resulted in a genome 54,721 bp in length with 203-bp direct terminal repeat ends and a GC content of 57.5%.

Using standard procedures (Pope et al., 2017), the software DNA Master v5.23.6 (http://cobamide2.bio.pitt.edu), PECAAN (https://discover.kbrinsgd.org), Genemark v2.5p (Lukashin and Borodovsky, 1998), and Glimmer v3.02 (Delcher et al., 1999) were used to predict 53 protein-encoding genes. Start sites were determined using Starterator v485 (https://seaphages.org/software/#Starterator) and Blastp v2.13.0 (Altschul et al., 1990) alignments against the Actinobacteriophage protein (Russell and Hatful, 2017) and NCBI non-redundant protein sequences databases (https://blast.ncbi.nlm.nih.gov). No strong evidence for tRNAs was found using Aragorn v1.2.41 (Laslett and Canback, 2004) and tRNAscan-SE v2.0 (Lowe and Eddy, 1997). A total of 14 genes were assigned putative functions using BLASTp v2.13.0 (Altschul et al., 1990), Phamerator (Cresawn et al., 2011), and HHpred (searching against PDB_mmCIF70, SCOPe70, Pfam-A, and NCBI_Conserved_Domains databases) (Söding et al., 2005). deepTMHMM v1.0.24 (Krogh et al., 2001) and SOSUI (Hirokawa et al. 1998) detected an additional 3 genes as membrane proteins. All software used default settings. The annotation is presented in Fig. 1B

KingKamren was assigned to the EK cluster using the GCS tool (Hirokawa et al., 1998), based on having a gene content similarity (GCS) of at least 35% to other EK bacteriophages in the Actinobacteriophage database. The EK cluster currently contains 56 members. The majority of EK phages are placed into one of two subclusters, EK1 or EK2, but KingKamren is one of three phages (to date) that are not sub-classified, as its GCS is similar to both EK1 and EK2 phages (Figure 1b). This small subset of EK phages share 4 genes (KingKamren’s genes 12, 26, 35, and 47 – Fig. 1C ) of unknown function that are unique to this group and do not share significant sequence similarity to any other actinobacteriophage in the database (phagesdb.org). KingKamren also has a 13,452 bp gene (gene 28, Fig. 1B ) which constitutes 24.6% of its entire genome and encodes a 4,483 amino acid protein of unknown function. This feature is found across all members in the EK phage cluster and represents one of the largest genes in actinobacteriophages (Jacobs-Sera et al., 2020).

**Nucleotide sequence accession numbers**

KingKamren is available at GenBank with Accession No. XPP978791 and Sequence Read Archive (SRA) No. SRX25029057.
